# Assessment of the coordination of integrated health service delivery networks by the primary health care: COPAS questionnaire validation in the Brazilian context

**DOI:** 10.1186/s12875-015-0299-5

**Published:** 2015-07-22

**Authors:** Ludmila Barbosa Bandeira Rodrigues, Claudia Benedita dos Santos, Sueli Leiko Takamatsu Goyatá, Marcela Paschoal Popolin, Mellina Yamamura, Keila Christiane Deon, Luis Miguel Veles Lapão, Marcelino Santos Neto, Severina Alice da Costa Uchoa, Ricardo Alexandre Arcêncio

**Affiliations:** Institute for Health Sciences, Federal University of Mato Grosso (UFMT), 1200 Alexandre Ferronato Avenue – Industrial Sector, 78557267 Sinop, MT Brazil; Mother-Baby Department, University of São Paulo at Ribeirão Preto, College of Nursing, 3900 Bandeirantes Avenue, 14040-902 Ribeirão Preto, SP Brazil; Nursing Department, Federal University of Alfenas, 700 Gabriel Monteiro da Silva St., 37130000 Alfenas, MG Brazil; Physical Education Department, Federal University of Rio Grande do Sul Physical Education College, 750 Felizardo St., Jardim Botânico, 90690-200 Porto Alegre, RS Brazil; WHO Collaborating Center for Health Workforce Policy and Planning, International Public Health and Biostatistics, Global Health and Tropical Medicine, Instituto de Higiene e Medicina Tropical, Universidade Nova de Lisboa, 100 Junqueira, Lisbon, 1349-008 Portugal; Public Health Department, Federal University of Rio Grande do Norte. Campus Universitário, Salgado Filho Avenue, Natal, RN Brazil

**Keywords:** Primary Health Care, Health Care Networks, Coordination, Psychometric analysis, Validation studies

## Abstract

**Background:**

Health systems organized as networks and coordinated by the Primary Health Care (PHC) may contribute to the improvement of clinical care, sanitary conditions, satisfaction of patients and reduction of local budget expenditures. The aim of this study was to adapt and validate a questionnaire - COPAS - to assess the coordination of Integrated Health Service Delivery Networks by the Primary Health Care.

**Methods:**

A cross sectional approach was used. The population was pooled from Family Health Strategy healthcare professionals, of the Alfenas region (Minas Gerais, Brazil). Data collection was performed from August to October 2013. The results were checked for the presence of floor and ceiling effects and the internal consistency measured through *Cronbach alpha*. Construct validity was verified through convergent and discriminant values following Multitrait-Multimethod (MTMM) analysis.

**Results:**

Floor and ceiling effects were absent. The internal consistency of the instrument was satisfactory; as was the convergent validity, with a few correlations lower then 0.30. The discriminant validity values of the majority of items, with respect to their own dimension, were found to be higher or significantly higher than their correlations with the dimensions to which they did not belong.

**Conclusion:**

The results showed that the COPAS instrument has satisfactory initial psychometric properties and may be used by healthcare managers and workers to assess the PHC coordination performance within the Integrated Health Service Delivery Network.

## Background

Population aging is now a universal phenomenon, being a characteristic of developed countries as well as, increasingly, of developing countries. Estimates show that by 2050, the planet will have approximately 2 billion elderly people, which will account for about 30 % of the global population. Forecasts suggest that by 2025 Brazil could reach the sixth place in the number of elderly people in the world; and in 2013 individuals aged 60 years or more already accounted for 12 % of the population [1].

The incidence of non-communicable chronic diseases increases with aging, including diabetes, hypertension, cancer and chronic obstructive respiratory diseases. According to the 2012 report of the Pan American Health Organization [2], these diseases accounted for approximately 35 million deaths in 2005, representing 60 % of all deaths in the world. It should be noted that chronic diseases, according to this report, generate high costs, especially in the health systems of developing countries, which have very limited resources [2]. These health service systems have been shown to be only slightly effective, as they are geared to acute conditions and organized in a fragmented way, with insufficient physical capacity and human resources to address chronic conditions, despite the full investment of financial resources [2, 3]. Thus, new forms of organization of these systems have been designed to better treat chronic conditions, aiming to be more equitable, cost-effective and to generate satisfaction and quality of life for their users.

With the prospect of re-orientation of these healthcare service systems, the Pan American Health Organization (PAHO) proposed an approach of Integrated Health Service Delivery Networks (IHSDNs) under the coordination of the Primary Health Care (PHC) [2, 4, 5].

IHSDNs are defined as a group of organizations that provide, or make arrangements to provide, equitable, integrated health services to a defined population. They are held accountable for the health status and clinical outcomes of the population served and to provide comprehensive services (high-quality, responsible and humanized care), covering all levels of prevention and care, and with coordination, integrating all care levels and settings through the Primary Health Care [2].

The IHSDN can be characterized by the establishment of horizontal relations between the care points and PHC, which is considered as the initial care point, emphasizing the problem-solving function of primary care in the most common health problems and based on which care is accomplished and coordinated in all care points [6].

Meaning that PHC, besides its function of solving more than 90 % of the most common health problems, should coordinate the health system [7].

Coordination by PHC should be understood as the ability to recognize and address the health problems of the populations, within their territories, and to promote integration among all health services the user requires, guaranteeing the integrality of care. Thus, making PHC responsible for integrating all care the patient receives, independently of where it is being delivered [8].

Therefore the integration of Health Service Delivery Networks by PHC involves the existence of a regular service, the constitution of PHC services as the preferred entry door, the guaranteed access to the different care levels and the coordination of actions, guaranteeing continuing care [9]. The integration of health systems should improve the system performance, so that the efforts are justified to the extent that they lead to more accessible and higher quality services with a better cost-benefit relation, and users satisfaction [10].

The IHSDN is an alternative to the traditional model, whereas the PHC though located in the first level of care, performs no coordination function.

**Fig. 1:**
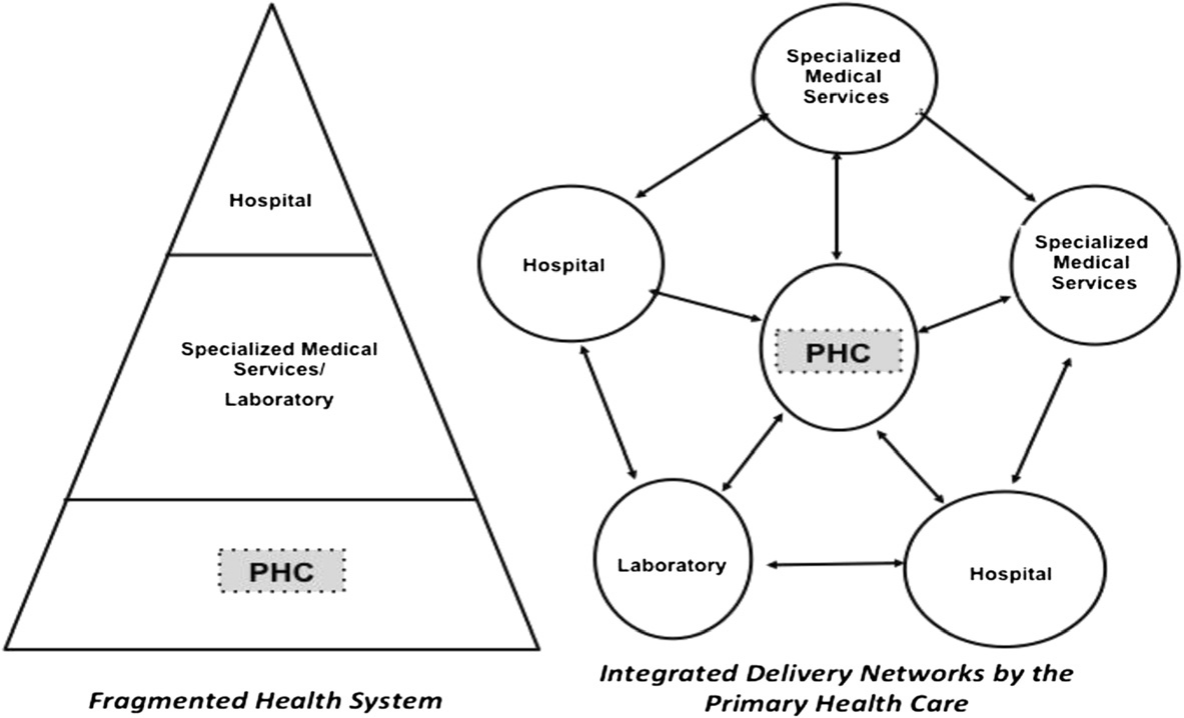
Fragmented and Health Care Networks schemes (Modified from Mendes, 2002 [11])

Figure 1 schematically presents the main differences between the current traditional model (fragmented) and the Healthcare Networks envisioned by PAHO [11]. In the Network conformation, the PHC is formally situated in the center, being an articulator between the other levels of the system. It is possible to observe a mobility of users through the health system according to their needs; however, this mobility will always be coordinated by PHC.

A recent study emphasized the importance of the PHC in coordinating the IHSDNs, highlighting it as blueprint strategy for the XXI century [12]. It must solve the majority of the health needs of the population and therefore to be able to cope with chronic conditions demand growth. Coordination is highlighted as a key attribute of the new configuration of the PHC, with lower costs, greater efficiency and resolutivity [12].

In Brazil, two types of PHC systems have been implemented; one includes gynecologists, pediatricians and general practitioners, nurses, auxiliary nurses and/or nursing technicians, with or without, Community Health Agents (CHA) and covers a defined geographical area. The other is the more recent Family Health Strategy (FHS), which consists of a general practitioner, a generalist nurse, auxiliary nurses and nursing technicians and CHAs. The latter covers a geographical area of reference, with a population of up to 4,000 inhabitants and its work should essentially be performed in the community through home visits [13]. Since 1996, the practice of the PHC has been particularly performed through the FHS, with this modality having a strategic and decisive role in coordinating the IHSDNs [14]. According laws and rules in Brazil the coordination of care should be conducted mainly through PHC and managers of the health local systems must provide all resources to ensure that and achieving universality, equity and comprehensive which are the principles of the Unified National Health System (SUS) [13].

Although the idea of IHSDNs is not new, having started in the mid-1920s in the UK with the Dawson Report [15], in Brazil, the proposal has grown stronger in recent years, especially after the recommendation by PAHO for the management of chronic conditions [2] and some evaluations regarding the health outcomes produced by the health systems in the country.

Despite the relevance of the theme for the Brazilian health system, instruments that can actually assess and measure the PHC’s ability to effectively coordinate an IHSDN are not observed in the literature. Some instruments have been validated in the Brazilian context to evaluate the attributes of the PHC, in general terms, such as the European Task Force on Patient Evaluation of General Practice Care (EUROPEP); the Primary Care Assessment Tool (PCATools) and the Program for Improving Quality and Access in Primary Care (PMAQ) [16, 17] however, despite containing some indicators that evaluate the quality of the PHC services, they do not include more specific and underlying aspects of the coordination of an IHSDN by the PHC.

Tool for Assessment of the Coordination of Integrated Health Service Delivery Networks by the Primary Health Care (COPAS) was the one that came closer to accomplishing this goal reason why the authors selected this tool. It widely has been used in the municipalities of the state of Minas Gerais to evaluate the level of development of IHSDNs. Then, in agreement with the original author [6], the COPAS was adapted and submitted to validation.

The tool is a comprehensive instrument built to audit the several health care management coordination dimensions, being them: Population, Primary Health Care, Support systems, Logistic systems and Management systems [6].

The expectation is that once the scientific validation is completed, the instrument can be used by managers and health workers for the situational diagnosis of their health systems and thus provide evidence regarding the rearrangements necessary to increase quality and efficiency. This study aimed to present the results of the pilot phase (phase I) of the validation of the COPAS to assess the coordination of IHSDNs by the Primary Health Care.

## Methods

### Study design

This was a cross sectional study [18, 19].

**Fig. 2:**
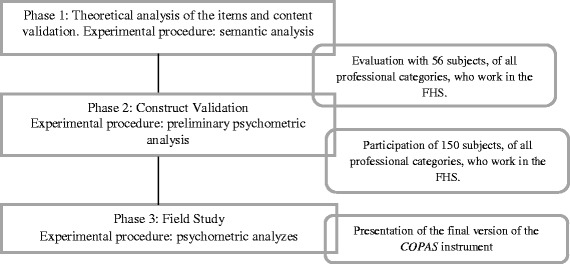
A general overview of the phases in the validation of the questionnaire to evaluate the Coordination of IHSDNs by the Primary Health Care (COPAS)

The validation of the instrument was composed of a sequence of phases, as described in Fig. 2. This paper presents the results derived from the pilot study of the instrument validation process (phase 2).

### Population sample and study location

The pilot study was conducted in the Alfenas microregion of Minas Gerais state. The region comprises part of the Southern area of the state and encompasses 11 municipalities, with a population of 201,119 inhabitants. The sample was pooled from local healthcare professionals working in units attached to the Family Health Strategy (FHS). Probabilistic, randomized and stratified sampling was used, proportional to each respective professional category, resulting in 150 subjects [20]; distributed among the professional categories as follows: 46 higher education professionals (physicians, nurses, dentists, physiotherapists, nutritionists, psychologists, and pharmacists); 104 high school education level professionals (Community Health Agents - CHA, Dental Assistants - DA, Auxiliary Nurses - AN, and Nursing Technicians - NT). The selection criterion was to have been employed in the health field for a minimum of six months. Those health professionals who were not active in their professional during the data collection period were excluded.

### Data collection

Data was collected from August to October 2013 in the health centers where the professionals worked. Previously trained interviewers were utilized, aiming to standardize the process of contact with the subjects. The instrument was self-administered.

### Questionnaire and measures

**Fig. 3:**
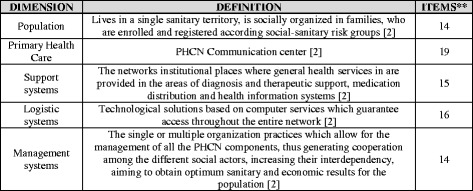
Definitions of the dimensions of the questionnaire and number of items in each dimension

The definition and number of items belonging to each dimension of the adapted instrument*,* named “*Tool for Assessment of the Coordination of Integrated Health Service Delivery Networks by the Primary Health Care - COPAS”* are presented in Fig. 3.

These dimensions represent essential conditions to qualify the PHC in the performance of coordination and should be highlighted in the IHSDN [2, 21]. Therefore, the idea of a robust PHC is one that is able to develop competences in these areas.

It should be highlighted that the instrument has a Likert type scale, with scores ranging from 1 to 5, with the following response options: 1, strongly disagree; 2, disagree; 3, neither agree nor disagree; 5 strongly agree. Thus implying the use of non-parametric statistics.

### Data analysis

The results were checked for the presence of floor (F) and ceiling (C) effects. The presence of F and C effects is considered in dimensions when more than 15 % of the subjects achieve the lowest or highest possible score in the instrument. The presence of F and C effects may indicate a loss in the responsiveness of the adapted instrument [22, 23].

*Cronbach alpha* [24] was used to measure the internal consistency of the adapted version of the instrument. *Alpha* values equal or superior to 0.70 were adopted as the reference [25].

The convergent and discriminant validities of the construct were analyzed using Multitrait-Multimethod analysis (MTMM explores the relationship of the item with the dimensions of the instrument) with the Multitrait Analysis Program (MAP) [26]. This method is especially useful when there is a large number of items, which leads to a considerable number of correlations [23, 27].

The validation of initial studies considers the convergent validity to be satisfactory if the linear correlation between an item and its dimension is higher than 0.30. For final studies, this value increases to 0.40. Discriminant validity is met when the linear correlation between the item and the dimension to which it hypothetically belongs is statistically higher than its correlation with the other dimensions [23, 27].

The Statistical Package for the Social Sciences program was used throughout the analysis. A significance level of 5 % (α =0.05) was adopted for the study. Data was double entered to ensure accuracy.

### Ethical aspects

The study was approved by the Research Ethics Committee of the University of São Paulo at Ribeirão Preto, College of Nursing, under authorization No. 10853412.4.0000.5393. All subjects read and signed the consent form and were assured of the confidentiality of the study.

## Results

The mean age of the participants was 33.5, the youngest being 22 and the oldest 65 years of age. The number of years in the job ranged from a minimum of 6 months to a maximum of 15 years; the mean was 4 years.

Table 1 presents the demographics characteristics according to the professional categories of the study participants.

**Table 1:** Characteristics of the participants according to their respective professions. Alfenas, Minas Gerais, 2013

Characteristics	Number	Percent
Gender		
Female	129	86.0
Male	21	14.0
Mean age in years/ Standard deviation	33.5/8.7	100.0
Professional Category		
Community Health Agent	79	52.6
Nursing technician or Auxiliary nurse	15	10.0
Nurse	15	10.0
Physician	15	10.0
Dental Assistant	10	6.7
Dentist	10	6.7
Physiotherapist	02	1.3
Nutritionist	02	1.3
Pharmacist	01	0.7
Psychologist	01	0.7
Mean time in the job/Standard deviation	4.0/3.4	100.0

Floor and ceiling effects were found to be absent in the dimensions, as a concentration above 15 % in the lowest and highest values was not found.

The instrument was validated using *Cronbach alpha* (Table 2).

**Table 2:** *Cronbach alpha* for each dimension when the item was excluded from COPAS

Population		PHC		Support systems		Logistic systems		Management	
Item	α*	Item	α*	Item	α*	Item	α*	Item	α*
01	0.63	15	0.78	34	0.85	49	0.71	65	0.79
02	0.65	16	0.77	35	0.86	50	0.69	66	0.77
04	0.63	18	0.77	36	0.86	51	0.72	67	0.79
05	0.63	19	0.76	37	0.86	52	0.70	68	0.78
06	0.60	20	0.75	38	0.86	53	0.70	69	0.78
07	063	21	0.76	39	0.86	54	0.76	70	0.79
08	0.62	22	0.77	40	0.87	55	0.71	71	0.79
09	0.64	23	0.77	41	0.85	56	0.73	72	0.85
10	0.65	24	0.75	42	0.85	57	0.72	73	0.79
11	0.67	25	0.75	43	0.85	58	0.73	74	0.79
12	0.67	26	0.75	44	0.85	59	0.71	75	0.80
13	0.63	27	077	45	0.86	60	0.71	76	0.79
14	0.66	28	0,75	46	0.85	61	0.69	77	0.82
		29	0.77	47	0.86	62	0.70	78	0.81
		30	0.75	48	0.85	63	0.69		
		31	0.77			64	0.73		
		32	0.76						
		33	0.78						
α dimension	0.65		0.78		0.86		0.73		0.81

α* = *Cronbach alpha* when the item was excluded

Table 2 shows that the *Cronbach alpha* values obtained in the COPAS dimensions ranged from 0.655 and 0.865. The ‘population’ and ‘support system’ dimensions had the lowest (0.655) and the highest (0.865) values, respectively. These values express the internal consistency, this being a satisfactory measure of the reliability.

Table 3 presents the convergent validity, according to the criterion adopted, which was found to be satisfactory for the majority of the items (the adopted criterion was a correlation between the item and the dimension to which it belongs of > 0.30 for initial studies).

**Table 3:** Convergent validity of COPAS obtained through Multitrait-Multimethod analysis. Alfenas, Minas Gerais, 2013

Population		PHC		Support systems		Logistic systems		Management	
Item	R	Item	R	Item	R	Item	R	Item	R
01	0.35	15	0.07	34	0.53	49	0.37	65	0.49
02	0.19	16	0.26	35	0.53	50	0.54	66	0.76
03	0.44	17	0.31	36	0.47	51	0.24	67	0.47
04	0.44	18	0.18	37	0.53	52	0.45	68	0.67
05	0.33	19	0.33	38	0.52	53	0.48	69	0.61
06	0.48	20	0.58	39	0.51	54	−017	70	0.56
07	0.32	21	0.39	41	0.56	55	0.37	71	0.53
08	0.37	22	0.27	42	0.63	56	0.18	72	−0.20
09	0.26	23	0.30	43	0.57	57	0.29	73	0.52
10	0.18	24	0.51	44	0.53	58	0.18	74	0.47
11	0.14	25	0.51	45	0.38	59	0.34	75	0.38
12	0.12	26	0.56	46	0.59	60	0.32	76	0.56
13	0.36	27	0.30	47	0.46	61	0.58	77	0.17
14	0.14	28	0.57	48	0.61	62	0.47	78	0.34
		29	0.18			63	0.52		
		30	0.45			64	0.09		
		31	0.28						
		32	0,35						
		33	0.14						

*r* Pearson’s linear correlation coefficient

Table 4 presents the discriminant validity and the percentage of items with higher and significantly higher correlations to their respective dimensions, rather than to other dimensions, evidencing positive adjustments in relation to the discriminant validity. The dimensions ‘support system’ and ‘management’ obtained 91.7 % and 89.3 %, respectively.

**Table 4:** Discriminant validity of COPAS according to Multitrait-Multimethod analysis. Alfenas, Minas Gerais, 2013

	Population		PHC		Support systems		Logistic systems		Management	
	n items	%	n items	%	n items	%	n items	%	n items	%
−2	0	0.0	4	5.3	0	0.0	7	10.9	4	7.1
−1	16	28.6	17	22.4	5	8.3	13	20.3	2	3.6
1	29	51.8	42	55.3	24	40.0	30	46.9	31	55.4
2	11	19.6	13	17.1	31	51.7	14	21.9	19	33.9
Adjustment		71.4		72.4		91.7		68.8		89.3

The values from −2 to 2 in the table correspond to the following: 2: the item correlation with the dimension to which it belongs is significantly higher than the correlation to the dimension to which it does not belong; 1: the item correlation to the dimension to which it belongs is higher than the correlation to the dimension to which it does not belong; −1: the item correlation to the dimension to which it belongs is lower than the correlation to the dimension to which it does not belong; −2: the item correlation to the dimension to which it belongs is significantly lower than the correlation to the dimension to which it does not belong.

## Discussion

Currently, there is great concern about the internal consistency of the instruments used to evaluate health systems. Thus, one should emphasize the importance of valorizing the operational steps in the adaptation and validation of an instrument, because, that way, the accuracy and quality of information collected and, consequently, its analysis can be guaranteed [28, 29].

In this context, the adaptation and validation of the COPAS instrument presents itself as a chance to contribute significantly to the strengthening of local health systems, as one of the difficulties that the majority of municipalities have faced is the lack of comprehension of the steps necessary to strengthen a health system, as well as experiencing, given the increasing chronic disease load, the challenges of overhauling the health system [30].

The generation of knowledge for “health system strengthening” requires qualified data, analytical skills and the ability to synthesize, adapt and disseminate evidence, therefore, the use of COPAS, can foster a culture based on scientific data, supporting decision making by managers aimed at developing sustainable health systems [30–32].

Among the tools that have been used in Brazil to assess PHC, the PCATools, EUROPEP and, more recently, the PMAQ are highlighted, the first of which being more widely used in Brazil. The EUROPEP was developed to provide *evidence on* improvements in the practice, performance and organization of family medicine professionals’ care in PHC. The PCATools measures the presence and extent of the four core attributes (First contact, Longitudinality, Integrality and Coordination) and the three derivatives of PHC (Family orientation, Community orientation and Cultural competency) and the user’s degree of affiliation with the health service.

The PMAQ aims to enhance the access and improve the quality of PHC, guarantee a national, regional and locally comparable quality standard with a view to greater transparency and effectiveness of governmental actions focused on PHC [17]. Although these instruments are used to assess PHC, none of them assesses PHC from the perspective of health care services coordination, which contains the support systems, logistic systems and governance systems as essential elements for the purpose of coordination.

In that sense, the COPAS can turn into an important tool for managers and other professionals to assess their system and the effectiveness of PHC as a coordinator, which can influence the definition of public policies and equity in health. COPAS’ validation study can contribute by completing an existing gap, which is the lack of validated instruments to measure the capacity of PHC to coordinate the IHSDN. Therefore, it is expected that, in the future, it can be used as a management tool, mainly in the almost six thousand Brazilian cities that want to make changes in their systems guided by PHC.

In face of these results, one can anticipate that the COPAS instrument can be used beyond the academic context, expecting its ability to prospect changes in the organization and management form of local health systems. The dimensions defining the instrument permit a more focused assessment of the constituent elements of an integrated system, the central position of the processes in the territories with a defined population assigned to PHC, the definition of user flows and counter-flows through the systems, the logistic and support systems that permit the integration among the units and governance systems with common goals and missions among all services in an IHSDN.

In methodological terms, it should be highlighted that, to assess the initial psychometric properties of the COPAS instrument, the absence of floor and ceiling effects was verified, in order to ensure the detection of extreme values in the items. The results indicate a high level of responsiveness for the instrument, an important characteristic to assure detection of changes in the future. According to Terwee et al. [25] in order to maintain the validity of the contents it is important to verify the absence of accumulation of answers in the floor and ceiling limits of the scale. If more than 15 % of the answers are concentrated within those limits, it may indicate losses concentrated at the extremes, which in turn may indicate limited content validity. This adapted version demonstrated a good level of content validity.

In the different dimensions of the study the *Cronbach alphas* ranged from 0.655 to 0.865, acceptable, good, very good or almost perfect values according to the criteria adopted [25].

The discriminant validity analyzed through Multitrait-Multimethod analysis demonstrated that the collinear relationship between the items and their respective dimensions (results for the majority of items) were higher or significantly higher than the relationship with the dimensions to which they did not belong. Such results confirm that COPAS possesses strong discriminant properties for the different dimensions. However, the expectation is that additional fieldwork (stage 3) will show better adjustments for the relative values belonging to the remaining dimensions.

Multitrait-Multimethod analysis was also used to analyze the convergent and discriminant validities in order to validate the construct. The ‘support system’ dimension validity was very satisfactory, obtaining a linear correlation of 100 %, with values above 0.30. These results are considered ‘ideal’ for initial studies [23]. Furthermore the initial psychometric properties of the study suggest that COPAS may be validated and reliable and its use may help in the search for solutions to the challenges faced by the PHC in becoming the coordinating axis for the IHSDNs.

The results presented in the article referred to the scale validation phase, with data obtained in the pilot phase (phase 2), which demonstrated satisfactory values. Thus, it is expected that, when the scale has been finally validated (phase 3), it may be used by managers and healthcare professionals to diagnosis the coordination of their systems, allowing weaknesses to be identified and improvements to be promoted, through recommendations, aiming to reorganize the systems in order to make them more equitable, efficient and resolutive.

It can be said that as soon as the World Health Organization advocates the importance of structuring the systems controlled by the PHC, other health systems in the world may also use the COPAS questionnaire, validating it for their contexts and cultures.

PHC managers may use the COPAS to assess their health services and identifying the critical aspects to develop and support the IHSDNs. This tool may be applied in health care professionals who are working in the PHC and its results may be discussed with these workers in workshops, meeting, seminars, among others. This tool also may be used in different moments to assess the degree of development of the IHSDNs coordinated by the PHC.

The managers can still consider this tool to establish goals in short, medium and long time and to adjust their system according IHSDNs. It is important to highlight that changing of health system is a complex and dynamic process and through the COPAS would be possible to define clearly the steps and time to achieve a good level of the IHSDNs.

As research limitations, it is highlighted that a convenience sample was selected. Nevertheless, as this was a pilot study, the sampling design did not entail any bias for the research results, as the idea in this phase is to test the psychometric properties of the instrument and its internal coherence. Another limitation is the number of items in the instrument, which can somehow create an information bias in the results, although the initial tool contained much more items and other tools the health services use, such as the PCTA tool, also include much more items than the instrument under analysis.

## Conclusion

The present study was developed following the proposed guidelines for the adaptation and validation of instruments for the quantitative measurement of subjective constructs. The initial results indicate that COPAS is a valid and reliable instrument and may, in the future, become widely used by researchers and healthcare workers as an instrument to audit and improved healthcare services coordination. However, the findings still need to be confirmed through fieldwork and with a larger sample. The fieldwork should include additional properties such as confirmatory factorial analysis.

Considering that a healthcare network which has the PHC as its organizing axis, needs the full commitment, work and participation of all healthcare professional and its various teams, it is suggested that COPAS may become a useful tool for professional involved in the reorganization of the health systems, as it promotes the professionals’ reflections in search of better care integration, in view of the fragmentation of health services.

It is highlighted that this task is neither easy nor free from individual and collective tensions, as it involves power aspects and professional and institutional interests. Nevertheless, innovation in the health management processes is both possible and urgent. Countless evidence exists that supply-based fragmented systems do not respond to the population’s health needs and that the effective implementation of the IHSDN, coordinated by PHC, is one way, as it means introducing new practices, new management instruments, in an integrated, efficient and effective manner [33].

## Abbreviations

PAHO: Pan American Health Organization
IHSDNs: Integrated Health Service Delivery Networks
PHC: Primary Health Care
CHA: Community Health Agent
FHS: Family Health Strategy
SUS: Unified National Health System
EUROPED: European Task Force on Patient Evaluation of General Practice Care
PCATools: Primary Care Assessment Tool
PMAQ: Program for Improving Quality and Access in Primary Care
COPAS: Coordination of Integrated Delivery Networks by the Primary Healthcare questionnaire
DA: Dental Assistant
AN: Auxiliary Nurses
NT: Nursing Technicians
F: Floor
C: Ceiling
MTMM: Multitrait-multimethod
MAP: Multitrait Analysis Program
NCRP: Nursing College of Ribeirão Preto
USP: University of São Paulo
